# Supplementary material for: Deconstruction of the Anisotropic Magnetic Interactions from Spin‐Entangled Optical Excitations in van der Waals Antiferromagnets
Source: Adv Sci (Weinh). 2025 Nov 7;13(2):e05834. doi: 10.1002/advs.202505834 (PMC12786320; doi:10.1002/advs.202505834)
Supplement: Supplementary file 1 — Supporting Information [file ADVS-13-e05834-s001.pdf]

Supplementary Information for *Deconstruction of the anisotropic magnetic interactions from spin-entangled optical excitations in van der Waals antiferromagnets*

Dipankar Jana,[1,2,*] Swagata Acharya,[3,†] Milan Orlita,[1,4] Clement Faugeras,[1]
Dimitar Pashov,[5] Mark van Schilfgaarde,[3] Marek Potemski,[1,6,7,‡] and Maciej Koperski[2,8,§]

[1] *LNCMI-EMFL, CNRS UPR3228, Univ. Grenoble Alpes, Univ. Toulouse, Univ. Toulouse 3, INSA-T, Grenoble and Toulouse, France*
[2] *Institute for Functional Intelligent Materials, National University of Singapore, 117544, Singapore*
[3] *National Renewable Energy Laboratory, Golden, CO, 80401 USA*
[4] *Institute of Physics, Charles University, Ke Karlovu 5, Prague, 121 16, Czech Republic*
[5] *King's College London, Theory and Simulation of Condensed Matter, The Strand, WC2R 2LS London, UK*
[6] *CENTERA, CEZAMAT, Warsaw University of Technology, 02-822 Warsaw, Poland*
[7] *Institute of High Pressure Physics, PAS, 01-142 Warsaw, Poland*
[8] *Department of Materials Science and Engineering, National University of Singapore, 117575, Singapore*

I. COMPUTATIONAL DETAILS FOR BAND STRUCTURE AND EXCITONIC PROPERTIES FOR $MnPS_3$ AND $NiPS_3$

The quasiparticle self-consistent GW approximation [S1, S2] is a self-consistent form of Hedin's GW approximation. In contrast to conventional GW implementations, QSGW modifies the charge density and is determined by a variational principle [S3]. A great majority of discrepancies in estimating bandgap and excitonic spectra in insulators originate from the omission of electron-hole interactions in the RPA polarizability. By adding ladders to the RPA, electron-hole effects are taken into account. Generating W with ladder diagrams has several consequences; most importantly, perhaps, screening is enhanced and W reduced. This in turn reduces fundamental bandgaps and also valence bandwidths. Agreement with experiment in both one-particle and two-particle properties is greatly improved. The theory and its application to a large number of both weakly and strongly correlated insulators are given in Ref. [S4]. The importance of self-consistency in both QSGW and QS$G\hat{W}$ for different materials has been explored [S5]. Here, QSGW and QS$G\hat{W}$ are both quasiparticle self-consistency methods, but the latter includes electron-hole effects in W, via ladder diagrams. Furthermore, by augmenting QSGW with DMFT where a local vertex for spin and charge fluctuations is built in, the fidelity of the theory can be further improved [S6] and spin-flip atomic multiplets can be captured.

In **Fig. S1(a,b)**, we show the optical absorption spectra computed for $MnPS_3$ and $NiPS_3$ bulk crystals from the combined approach of QS$G\hat{W}$ and DMFT. Further, we show the real-space plots of the wavefunctions for the on-site spin-flip excitonic states (2.64 eV in $MnPS_3$ and 1.47 eV in $NiPS_3$) and the spin-allowed delocalized excitonic states (3.2 eV in $MnPS_3$ and 1.8 eV in $NiPS_3$). The spin-flip transition in both materials is primarily localized on the metal site, whereas the wavefunctions of spin-allowed transitions extend across multiple metal and ligand sites. In $MnPS_3$, the majority spin sector is fully occupied, preventing any on-site spin-allowed transitions. As a result, the 3.2 eV exciton transition is attributed to either *pd* or intersite *dd* character. In contrast, $NiPS_3$ allows on-site spin-allowed transitions, with the lowest-energy excitonic transition occurring at 1 eV, localized at the Ni atom.

* dipankar.jana@lncmi.cnrs.fr
† swagata.acharya@nrel.gov
‡ marek.potemski@lncmi.cnrs.fr
§ msemaci@nus.edu.sg

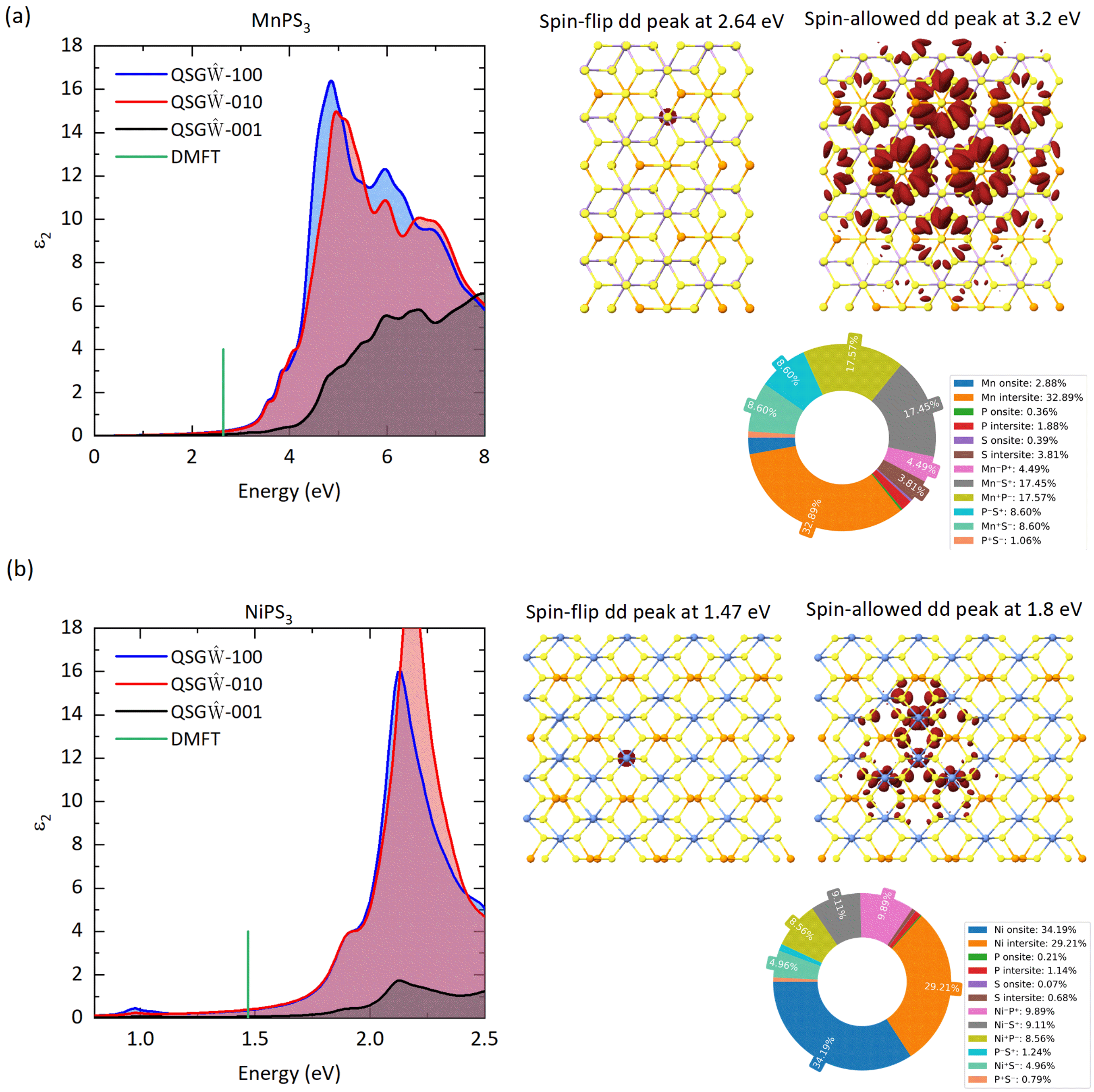

FIG. S1. Calculated optical absorption spectra along different polarization directions for **(a)** $MnPS_3$ and **(b)** $NiPS_3$. The spatial distribution of the exciton wavefunction for the on-site spin-flip and lowest energy spin-allowed transitions is also shown in the corresponding plots. Note that the lowest energy spin allowed transition in $MnPS_3$ (at 3.2 eV) is intersite, while it is on-site for $NiPS_3$ (at 1 eV). Here we present the spatial distribution of the wavefunction for the spin-allowed sub-bandgap feature at 1.8 eV in $NiPS_3$.

II. COUPLING OF THE POLARIZATION OF SPIN-FLIP TRANSITION WITH MAGNETIC ORDERING

For $NiPS_3$, the X-transition is reported to be linearly polarized due to its coupling with antiferromagnetic ordering [S7]. The in-plane magnetic field induces spin rotation, which is reflected in the rotation of the linear polarization axis of this spin-flip transition, as shown in **Fig. S2(a)**. The corresponding magnetic field-dependent splitting of the X-transition, presented in the main text, reveals two distinct components, indicating a single-domain flake. This is further supported by the high degree of linear polarization (DoLP) observed in the X-transition at 0 T as shown in

Fig. S2(a). The polarization axis is perpendicular to the spin direction [S7], meaning it is approximately perpendicular to the magnetic field (while the easy spin axis is nearly parallel). As the magnetic field increases, the polarization axis rotates and eventually aligns along the field direction (i.e., the spins align perpendicular to the field direction). Notably, both split components exhibit a significantly higher degree of linear polarization when spectrally resolved from small components appearing from the domain with different orientations of the easy spin axis. This is further examined with a multidomain sample. At 0 T, as shown in **Fig. S2(b)**, the DoLP is found to be far less than the previous sample. In this flake, the X-transition primarily splits into two sets of symmetric components, as shown by the false color plot in **Fig. S2(b)**, and all the split components have much higher DoLP. For one domain, the DOLP axis is nearly perpendicular to the field, leading to larger field-dependent splitting than in the other domains at an intermediate angle to the magnetic field. Far above the spin-flop field, all the domains align perpendicular to the field direction. At 30 T, we observe a loss in DoLP in both samples, indicating depolarization of the X-transition in the canted phase. This is possible if the polarization characteristics are entirely dictated by antiferromagnetic ordering.

In $MnPS_3$, the spins are aligned perpendicular to the plane. According to Ref. [S7], the X-transition in $MnPS_3$ is expected to exhibit circular polarization. We do not observe any linear polarization selectivity of the X-transition in $MnPS_3$. Since these transmission measurements were conducted in reflection geometry, the double transmission along opposite directions prevented us from isolating the split components of the X-transition using circular polarization-resolved measurements. Above the spin-flop field, the spins reorient along a specific in-plane axis (as there is a negligibly small in-plane anisotropy also [S8]), and based on Ref. [S7], the X-transition should then become linearly polarized. However, we do not observe any measurable linear polarization of X-transition above B_{sf}. This observation raises fundamental questions about how polarization couples to the spin direction.

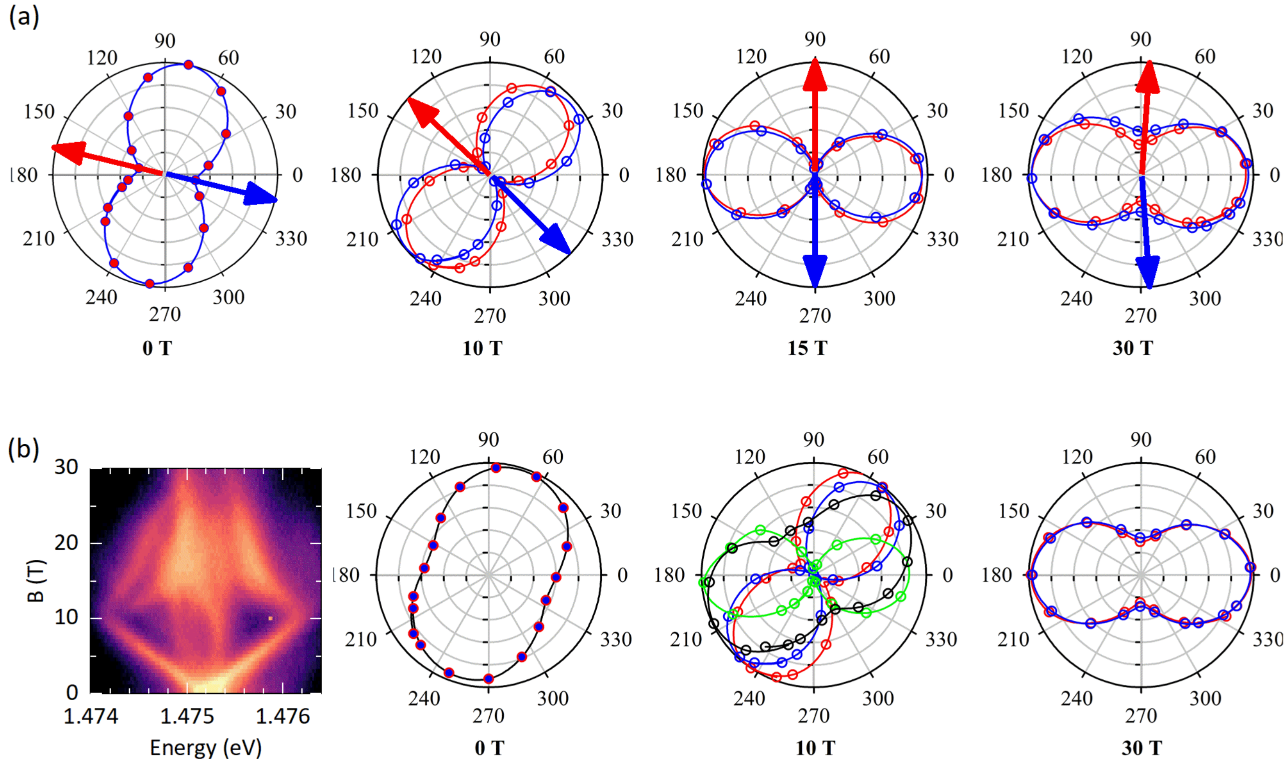

FIG. S2. **(a)** Polar plots of linear polarization resolved integrated photoluminescence intensity of the split components of X-transition of $NiPS_3$ at different magnetic fields applied in the in-plane direction (the corresponding photoluminescence spectra are presented in the form of a false color map in **Fig. 4d** of the main text). The direction of the spin sublattices is shown by blue and red arrows at different magnetic fields. A contribution of Faraday rotation induced by the objective lens has been subtracted, and the polarization orientation at $B = 30$ T was taken as a reference and set as 0°. One can notice that the zero-field angle between the magnetic field and the crystal's a-axis appears around 10° - 20°, which is consistent with the simulation in the main text where $\Psi(B) = 15°$ is considered. **(b)** False color photoluminescence map of a multidomain $NiPS_3$ sample and the polar plots at different magnetic fields measured under identical experimental conditions.

III. QUADRATIC MAGNETIC FIELD DEPENDENCE OF SPIN-FLIP TRANSITION AND ITS REPLICAS

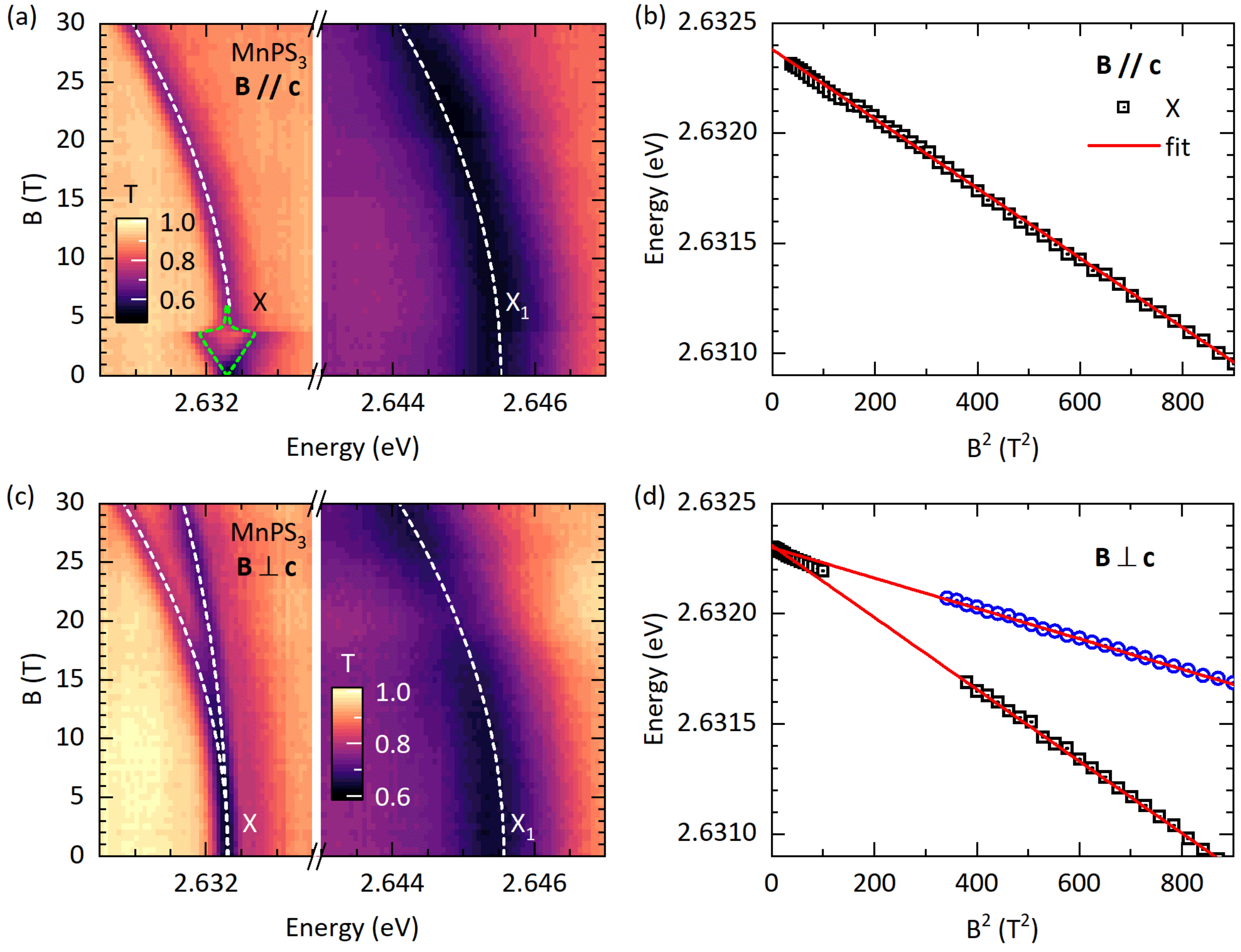

FIG. S3. Low temperature (5 K) false color map of the transmission spectra of $MnPS_3$ as a function of the magnetic field applied **(a)** along the easy spin axis and **(c)** perpendicular to the easy spin axis. **(b,d)** Corresponding magnetic field dependence of X-transition as a function of the square of the magnetic field. The red line shows a linear fit to the magnetic field dependence.

The in-plane and out-of-plane magnetic field dependence of the X-transition of $MnPS_3$ is presented in **Fig. S3(a,c)**, while the peak energy of the X-transition (including its split components) as a function of the square of the magnetic field is shown in **Fig. S3(b,d)**, respectively. A linear fit to the magnetic field dependence, indicated by the red line, yields a slope of $\frac{g^2\mu_B^2}{2S_gJ}$, as can be obtained from Eq. (1,2) of the main text, from which the exchange constant is calculated to be J=1.6 meV. For $B \perp c$-axis, the X-transition splits and both the split components exhibit a quadratic magnetic field dependence, as shown in **Fig. S3(d)**. The quadratic magnetic field dependence of the low energy split component is identical to the previous configuration (above B_{sf}), while the origin for the quadratic shift of the high energy split component remains unclear. Interestingly, the high-energy side band (X_1) follows identical quadratic magnetic field dependence without any splitting for both magnetic field orientations. This X_1 transition has been attributed to an excitonic transition coupled with magnon excitation from the higher-momentum magnon flat band region. Raman scattering measurements provide evidence of this magnon continuum excitation in the form of a two-magnon continuum band (2M), with an energy approximately twice that of a single-magnon excitation. As evidenced in Raman scattering measurements, the 2M resonance remains independent of the magnetic field. The energy spacing between the spin-flop exciton (X-transition) and the magnon sideband (X_1 transition) thus remains constant as a function of the magnetic field. Consequently, the X_1 transition exhibits the same magnetic field dependence as the X transition. However, a key unresolved aspect is why the X_1 transition does not split below the spin-flop field for the $B \parallel c$-axis configuration, even though the X transition does. This issue will be further discussed in the subsequent

section.

The false color PL map of $NiPS_3$ as a function of the square of the magnetic field applied perpendicular to the easy spin-axis is shown in **Fig. S4(a)**. A linear simulation of the low-energy split component, represented by the dashed line, allows for the estimation of the exchange constant as J=22 meV. To better illustrate the splitting, we show the Lorentzian profile fit to the X-transition at 0 T and the split components of the X-transition at 30 T in **Fig. S4(b)**. The effective exchange parameter, estimated for these two materials, closely matches the reported value ($J=z_1J_1+z_2J_2+z_3J_3$), where z_i and J_i correspond to the number of ith nearest neighbor and exchange interaction with i^{th} nearest neighbor, respectively. The interlayer exchange interaction is reported to be negligible compared to the intralayer exchange interactions, and thus it was not taken into account in the estimations of the interaction parameters from the magneto-optical spectroscopy data.

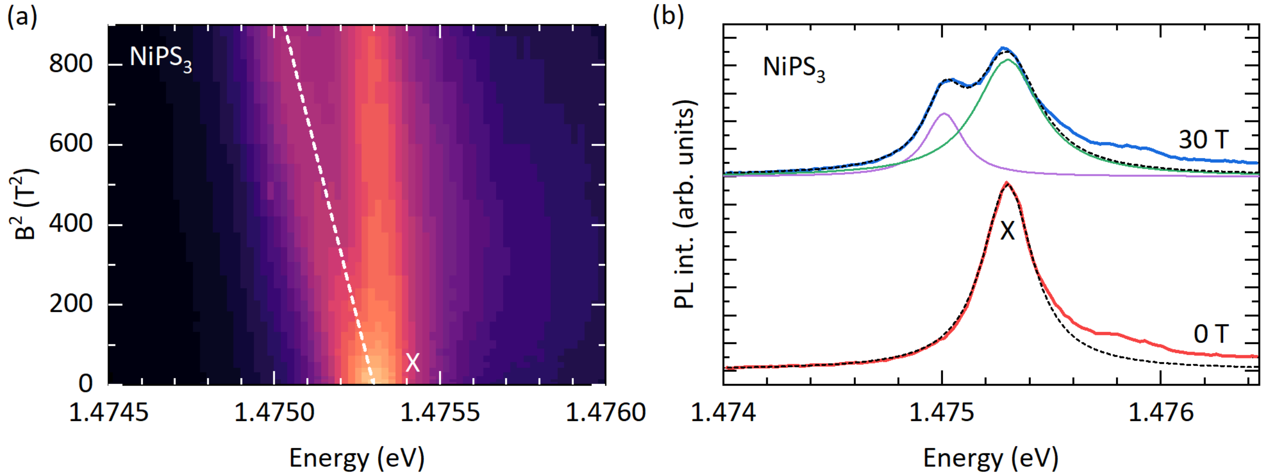

FIG. S4. **(a)** Low temperature (5 K) false color map of the photoluminescence spectra of $NiPS_3$ as a function of the square of the magnetic field applied perpendicular to the easy spin axis. The white dashed line corresponds to the simulated linear dependence of the low-energy split component. **(b)** 5 K photoluminescence spectra of $NiPS_3$ measured at 0 T and 30 T. A fit to the X-transition and its split components with a Lorentzian profile is also shown.

IV. COMMENTS ON THE CHARACTERISTICS OF X AND X_1 TRANSITION

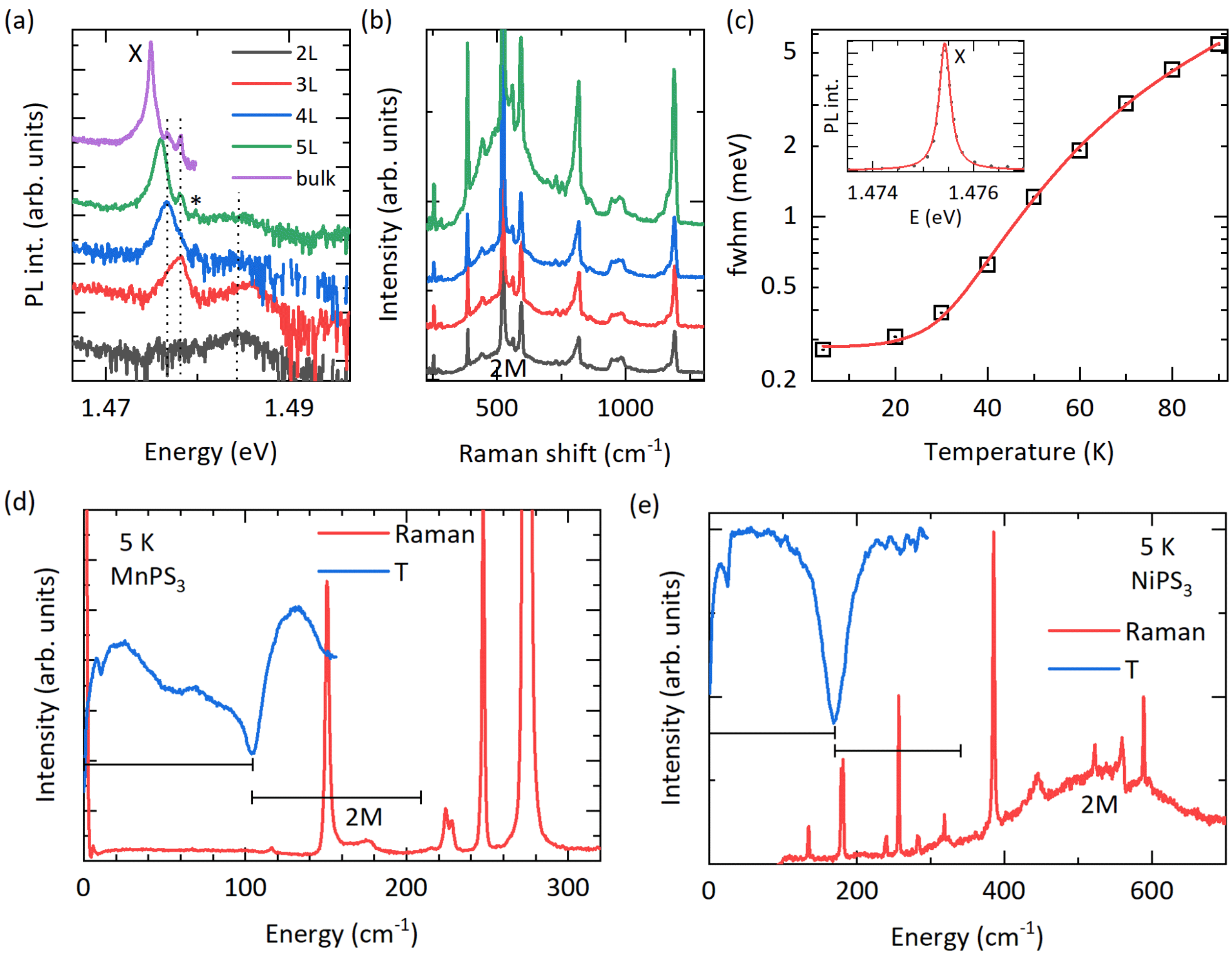

FIG. S5. Low temperature **(a)** PL and **(b)** Raman scattering spectra of $NiPS_3$ layers. '*' is the instrument response and not related to any PL from the material. **(c)** The width of the X-transition as a function of temperature. The inset shows the fit of a Lorentzian function to the X-transition. Low-temperature Raman scattering spectra (red curve) of **(d)** $MnPS_3$ and **(e)** $NiPS_3$. The two-magnon continuum transition is labeled as 2M. For comparison, the reflectivity spectrum is plotted as a function of energy with respect to the X-transition.

The sharp optical transition has been primarily interpreted through two competing scenarios: (1) the Zhang-Rice (ZR) picture [S9–S12], where the electron and hole are shared between the Ni 3*d* and ligand S *sp* orbitals; and (2) the atomic-multiplet model, where the exciton originates from an on-site *dd* transition [S13, S14]. Our theoretical framework (ED-DMFT) is constrained to modeling on-site spin-flip *dd* transitions and does not account for possible extensions of the exciton wavefunction onto surrounding ligand atoms. Nevertheless, it predicts spin-flip exciton energies in close agreement with experimental observations for both $NiPS_3$ and $MnPS_3$. Various theoretical approaches that either support or challenge the ZR scenario agree that a spin-flip process is involved in these sharp transitions. Here, we comment on few characteristics of this sharp transition in $NiPS_3$ that have been previously interpreted as evidence for the ZR exciton.

1. Dependence on antiferromagnetic ordering: A strong spin–charge coupling becomes accessible in the antiferromagnetic phase, which leads to the increased oscillator strength of the ZR optical transition below the Néel temperature [S10]. This explains the emergence of the 1.47 eV transition below T_N, accompanied by its strong intensity, despite being spin-forbidden. The reduction in the intensity of the X-transition with decreasing layer number, particularly its absence in the monolayer regime, is attributed to the softening of magnetic ordering with decreasing layer number [S15]. As shown in **Fig. S5(a,b)**, the X-transition persists down to the bilayer films, and the corresponding Raman scattering spectra reveal the presence of the two-magnon continuum (2M), indicating that

magnetic correlations remain intact down to the bilayer. Additionally, recent experimental work has reported the presence of magnetic ordering even in monolayer $NiPS_3$ samples [S16]. Our theoretical calculations also predict the existence of this transition in the monolayer. This is expected, as these excitons predominantly arise from electronic excitations localized on transition metal ions. The spatial extension of the wavefunction is insufficient to induce significant interlayer coupling. Therefore, the reduction in PL intensity with reducing layer number and its absence in monolayer $NiPS_3$ is expected to have a different origin.

2. Oscillator strength of X-transition and splitting in magnetic field: In the Zhang-Rice (ZR) exciton framework, the X-transition is interpreted as a spin-flip process between the ZR triplet state (S= ± 1) and the ZR singlet state (S=0) [S10]. Since such an electric dipole transition is governed by the spin selection rule ΔS=0, a simultaneous excitation of two spin sites with opposite spin orientations (S = +1 and S = −1) is considered for this optical process. Consequently, the oscillator strength of this transition becomes significant only in the antiferromagnetic phase. The observed splitting under a magnetic field applied along the crystal a-axis is attributed to the change in spin quantum number $\Delta m_S = \pm 1$ between ground and excited states with the same g-factors [S17]. Alternatively, Ref. [S11] proposes that it is the conservation of total angular momentum, rather than spin alone, that leads to the strong oscillator strength. The total angular momentum remains unchanged during the ZR excitation/emission process, rendering the transition optically bright. The observed splitting arises from ground and excited states with the same total angular momentum but distinct g-factors. Whether this interpretation can be generalized to other antiferromagnetic systems [S18, S19], where the splitting exhibits nearly identical slopes, remains an open question.

3. Saturation of the linewidth and its interpretation as exciton condensation: The spin-flip exciton is largely localized to the metal ion (in ED-DMFT calculations). As the wavefunction of this exciton is largely localized, the inhomogeneity-induced broadening is expected to be smaller than in the case of extended wave functions. It also does not involve phonon states from the ligand. This could be the reason for the narrow linewidth of the X-transition. However, considering the reported decay time of 10 ps [S20], the intrinsic width is approximately 37 μeV, which is much lower than the experimentally reported linewidth. Thus, even though the transition is narrow, it still has a small degree of broadening from thermal fluctuations or spatial inhomogeneity. Refs. [S10, S11] report that the linewidth of the X-transition saturates to 0.4 meV at low temperatures, which was interpreted as a signature of exciton condensation. However, as shown in the inset of **Fig. S5(c)**, we observe a significantly narrower linewidth of 0.26 meV, indicating that previous observations may include inhomogeneous broadening due to sample nonuniformity. Moreover, the linewidth increases monotonically with temperature (see **Fig. S5(c)**), consistent with thermal broadening. Fitting the temperature dependence, with equation: $\gamma = \Gamma_0 + \Gamma_1 \left(\exp\left(\frac{E_{LO}}{kT}\right) - 1\right)^{-1}$, yields a phonon energy E_{LO}=16 ± 1 meV (128 ± 8 cm^{-1}), which closely matches the lowest-energy phonon mode at 134 cm^{-1} (**Fig. S5(e)**). This coupling to phonons suggests that linewidth saturation cannot be taken as conclusive evidence for condensation.

The X_1 transition in $MnPS_3$ has been identified as the exciton-magnon continuum state, as the energy difference to the X-transition closely matches with half the energy of the two-magnon continuum band observed in Raman scattering measurements [S18]. Additionally, the absence of X_1 splitting below the spin-flop field has been explained by a linear magnetic field-dependent splitting of the magnon continuum transition. However, no magnetic field dependence of the 2M feature was observed in Raman scattering measurements. The $k = 0$ magnon gap is magnetic field dependent [S8], but its energy (0.5 meV) is significantly lower than the 13 meV spacing between the X and X_1 transition. Further, as shown in Fig. S5, the half-energy of 2M mode and its width does not match with the energy of the X_1 transition relative to the X-transition. These discrepancies call for a more thorough justification of whether X_1 should be considered an exciton-magnon continuum replica or if it originates from a different mechanism.

[S1] M. van Schilfgaarde, T. Kotani, and S. Faleev, "Quasiparticle self-consistent GW theory," *Physical Review Letters*, vol. 96, no. 22, p. 226402, 2006.

[S2] D. Pashov, S. Acharya, W. R. L. Lambrecht, J. Jackson, K. D. Belashchenko, A. Chantis, F. Jamet, and M. van Schilfgaarde, "Questaal: a package of electronic structure methods based on the linear muffin-tin orbital technique," *Comp. Phys. Comm.*, vol. 249, p. 107065, 2020.

[S3] S. Ismail-Beigi, "Justifying quasiparticle self-consistent schemes via gradient optimization in Baym–Kadanoff theory," *Journal of Physics: Condensed Matter*, vol. 29, no. 38, p. 385501, 2017.

[S4] B. Cunningham, M. Grüning, D. Pashov, and M. Van Schilfgaarde, "QSGŴ: Quasiparticle self-consistent GW with ladder diagrams in W," *Physical Review B*, vol. 108, no. 16, p. 165104, 2023.

[S5] S. Acharya, D. Pashov, A. N. Rudenko, M. Rösner, M. van Schilfgaarde, and M. I. Katsnelson, "Importance of charge self-consistency in first-principles description of strongly correlated systems," *npj Computational Materials*, vol. 7, no. 1, pp. 1–8, 2021.

[S6] S. Acharya, D. Pashov, C. Weber, M. van Schilfgaarde, A. I. Lichtenstein, and M. I. Katsnelson, "A theory for colors of strongly correlated electronic systems," *Nature Communications*, vol. 14, p. 5565, Sept. 2023.

[S7] X. Wang, J. Cao, Z. Lu, A. Cohen, H. Kitadai, T. Li, Q. Tan, M. Wilson, C. H. Lui, D. Smirnov, *et al.*, "Spin-induced linear polarization of photoluminescence in antiferromagnetic van der Waals crystals," *Nature Materials*, vol. 20, no. 7, pp. 964–970, 2021.

[S8] M. Kobets, K. Dergachev, S. Gnatchenko, E. Khats' ko, Y. M. Vysochanskii, and M. Gurzan, "Antiferromagnetic resonance in $Mn_2P_2S_6$," *Low Temperature Physics*, vol. 35, no. 12, pp. 930–934, 2009.

[S9] F. C. Zhang and T. M. Rice, "Effective hamiltonian for the superconducting Cu oxides," *Phys. Rev. B*, vol. 37, pp. 3759–3761, Mar 1988.

[S10] S. Kang, K. Kim, B. H. Kim, J. Kim, K. I. Sim, J.-U. Lee, S. Lee, K. Park, S. Yun, T. Kim, *et al.*, "Coherent many-body exciton in van der waals antiferromagnet $NiPS_3$," *Nature*, vol. 583, no. 7818, pp. 785–789, 2020.

[S11] F. Song, Y. Lv, Y.-J. Sun, S. Pang, H. Chang, S. Guan, J.-M. Lai, X.-J. Wang, B. Wu, C. Hu, *et al.*, "Manipulation of anisotropic Zhang-Rice exciton in $NiPS_3$ by magnetic field," *Nature Communications*, vol. 15, no. 1, p. 7841, 2024.

[S12] C. A. Belvin, E. Baldini, I. O. Ozel, D. Mao, H. C. Po, C. J. Allington, S. Son, B. H. Kim, J. Kim, I. Hwang, *et al.*, "Exciton-driven antiferromagnetic metal in a correlated van der Waals insulator," *Nature communications*, vol. 12, no. 1, pp. 1–7, 2021.

[S13] W. He, Y. Shen, K. Wohlfeld, J. Sears, J. Li, J. Pelliciari, M. Walicki, S. Johnston, E. Baldini, V. Bisogni, *et al.*, "Magnetically propagating Hund's exciton in van der Waals antiferromagnet $NiPS_3$," *Nature Communications*, vol. 15, no. 1, p. 3496, 2024.

[S14] I. Hamad, C. Helman, L. Manuel, A. Feiguin, and A. Aligia, "Singlet polaron theory of low-energy optical excitations in $NiPS_3$," *Physical Review Letters*, vol. 133, no. 14, p. 146502, 2024.

[S15] K. Kim, S. Y. Lim, J.-U. Lee, S. Lee, T. Y. Kim, K. Park, G. S. Jeon, C.-H. Park, J.-G. Park, and H. Cheong, "Suppression of magnetic ordering in XXZ-type antiferromagnetic monolayer $NiPS_3$," *Nature Communications*, vol. 10, no. 1, p. 345, 2019.

[S16] L. Hu, H.-X. Wang, Y. Chen, K. Xu, M.-R. Li, H. Liu, P. Gu, Y. Wang, M. Zhang, H. Yao, *et al.*, "Observation of a magnetic phase transition in monolayer $NiPS_3$," *Physical Review B*, vol. 107, no. 22, p. L220407, 2023.

[S17] X. Wang, Q. Tan, T. Li, Z. Lu, J. Cao, Y. Ge, L. Zhao, J. Tang, H. Kitadai, M. Guo, *et al.*, "Unveiling the spin evolution in van der Waals antiferromagnets via magneto-exciton effects," *Nature Communications*, vol. 15, no. 1, p. 8011, 2024.

[S18] S. Gnatchenko, I. Kachur, V. Piryatinskaya, Y. M. Vysochanskii, and M. Gurzan, "Exciton-magnon structure of the optical absorption spectrum of antiferromagnetic $MnPS_3$," *Low Temperature Physics*, vol. 37, no. 2, pp. 144–148, 2011.

[S19] J. Van der Ziel, "Optical spectrum of antiferromagnetic Cr_2O_3," *Physical Review*, vol. 161, no. 2, p. 483, 1967.

[S20] K. Hwangbo, Q. Zhang, Q. Jiang, Y. Wang, J. Fonseca, C. Wang, G. M. Diederich, D. R. Gamelin, D. Xiao, J.-H. Chu, *et al.*, "Highly anisotropic excitons and multiple phonon bound states in a van der Waals antiferromagnetic insulator," *Nature Nanotechnology*, vol. 16, no. 6, pp. 655–660, 2021.
